# Oxidative stress in occupational exposure to styrene vapors and dangerous chemicals in the shipbuilding industry

**DOI:** 10.3389/ftox.2023.1319896

**Published:** 2023-11-22

**Authors:** Daniela Pigini, Enrico Paci, Rachele Guglielmetti, Giovanna Tranfo, Mariangela Spagnoli, Annarita Fetoni, Laura Tricarico, Renata Sisto

**Affiliations:** ^1^ INAIL, National Institute for Insurance against Accidents at Work, Department of Occupational and Environmental Medicine, Epidemiology and Hygiene, Rome, Italy; ^2^ Department of Chemistry, University of Rome Sapienza, Rome, Italy; ^3^ Department of Neuroscience, Reproductive and Odontostomatological Sciences-Audiology Section Naples, University of Naples Federico II, Naples, Italy; ^4^ Catholic University of the Sacred Hearth, Faculty of Medicine and Surgery, Rome, Italy

**Keywords:** occupational exposure, mandelic acid, phenylglyoxylic acid, oxidative stress biomarkers, biological monitoring

## Abstract

**Introduction:** In the shipbuilding industry, workers are exposed to a variety of dangerous chemicals, styrene being one of them. The International Agency for Research on Cancer classified styrene as a chemical belonging to group 2A, which means it is probably carcinogenic to humans. This study aimed at evaluating the oxidative stress effects due to occupational exposure to styrene and other chemicals.

**Materials and methods:** Styrene urinary metabolites, such as mandelic acid and phenylglyoxylic acid, and the urinary biomarkers of oxidative stress, i.e., oxidation products of DNA and RNA and of proteins, were measured in a group of 17 workers and compared to the concentrations found in a group of 17 healthy volunteers who had not been exposed to chemicals.

**Results and discussion:** Statistically significant differences were found for 8-oxo-7,8-dihydroguanine (8-oxoGua) and 8-oxo-7,8-dihydro-2′-deoxiguanosine (8-oxodGuo) concentrations that are higher in workers than in the control group. The workers performing the tasks of painting are the most exposed to styrene and show higher concentrations of 8-oxo-7,8-dihydroguanosine (8-oxoGuo). Workers performing the tasks of wood refining and welding are less exposed to styrene but have higher concentrations of 8-oxoGua and 8-oxodGuo.

**Conclusion:** The exposure scenario in shipbuilding is a complex one, in which different xenobiotics are simultaneously present. The oxidative stress effect biomarkers, obtained from the oxidation product of RNA and DNA, are promising, sensitive, but not specific.

## 1 Introduction

The shipbuilding industry is a dynamic and competitive sector and is one of the oldest production industries, important from both an economic and social perspective. The production processes of shipyards are divided into two categories: the new shipbuilding and ship repair industry, both representing workplaces at a high risk of occupational diseases. The processes involve surface preparation, welding, painting, fiberglass manufacturing, molding, and solvent cleaning. All these involve the exposure of workers to several chemicals, such as degreasers, solvents, abrasives, and styrene vapors. The main health risk is associated to exposure to styrene vapors, solvents, and wood dust. Finally, exposure to fumes resulting from metal combustion during welding and cutting processes is also possible (Ünsalan and Celebi, 2010; Makarova and Tkalich, 2021).

Styrene is an aromatic hydrocarbon widely used in the production of plastics and used in the fiberglass-reinforced plastic industry, owing to the great reactivity of the vinyl double bond which makes it easily polymerizable and co-polymerizable, even at room temperature, but more rapidly at high temperatures (Rueff et al., 2009). The highest exposure to styrene was measured in occupational settings, particularly in the fabrication of reinforced plastic products (Paci et al., 2013; Kim, 2015; Banton et al., 2019). Styrene exposure occurs mainly through the inhalation of its vapors during lay-up or spray-up operations and lamination and curing steps (Lees et al., 2003; Rueff et al., 2009) and, to a minor extent, via skin contact (Costa et al., 2012). Exposure to styrene causes adverse effects in the peripheral and central nervous system, irritation of the skin and respiratory system, and mild liver damage; it is immediately absorbed through the skin and via the lungs, distributed in adipose tissues, and metabolized in the body. The International Agency for Research on Cancer (IARC) classified styrene as a chemical that is probably carcinogenic to humans, group 2A (IARC, 2019).

In humans, styrene is oxidized by cytochrome P-450-(CYP) into 7,8-styrene oxide (SO) which is considered to be directly responsible for the genotoxic effect. Then, SO is hydrolyzed by microsomal epoxide hydrolase (mEH) in phenyl ethylene glycol which is further metabolized to mandelic acid (MA) and phenylglyoxylic acid (PGA) (Carbonari et al., 2015).

MA and PGA represent 85% and 10% of the total amount of absorbed styrene excreted in urine, respectively (ACGIH, 2001). The dose biomarkers suggested by the American Conference of Governmental Industrial Hygienists (ACGIH) for occupational exposure are MA + PGA, with a BEI of 400 mg/g of creatinine or 0.2 mg/L of styrene in venous blood, both measured at the end of the work shift, corresponding to a threshold limit value–time-weighted average (TLV–TWA; concentration limit for a normal 8-h workday and a 40-h workweek to which nearly all workers may be repeatedly exposed, day after day, without adverse effects) of 85 mg/m^3^ which is equivalent to 20 ppm (ACGIH, 2020).

As previously stated, in addition to styrene, shipbuilding industry workers are also exposed to other dangerous volatile organic compounds (VOCs), some of which, according to the IARC, have both neurotoxic and carcinogenic properties. Benzene is classified by the IARC in group 1 (IARC, 2018), xylene and toluene are classified in group 3, and ethylbenzene is classified in group 2B (IARC, 2000; Cheng et al., 2019). To protect the health of workers, the airborne concentration levels of VOCs must comply with the occupational exposure limits (OELs) and the appropriate personal protective equipment must be worn if needed (Sisto et al., 2020).

In the shipbuilding industry, workers are also exposed to wood dust as carpentry activities are carried out. Wood dust comprises cellulose and other substances that depend on wood species. The size of the particles and their quantities depend on the operations during wood processing (Vallières et al., 2015).

Wood dust is classified by the IARC as carcinogenic, group 1, to humans for cancers of the nasal cavity, paranasal sinus, and nasopharynx, with an association between this type of cancer and exposure to hardwood dust (IARC, 2012). Exposure to hardwood dust may cause respiratory symptoms and diseases, the most serious health effect being the risk of nasal and sinonasal cancer. According to the Directive (EU) 2017/2398 (Directive EU, 2017/2398, 2017), the limit values for occupational exposure to hardwood dusts is 3 mg/m^3^ until 17 January 2023, and then, it will be reduced to 2 mg/m^3^. If hardwood dusts are mixed with other wood dusts, the limit value applies to all wood dusts present in that mixture.

Oxidative stress involves oxidative damage to nucleic acids, lipids, and proteins. Reactive oxygen species (ROS) and reactive nitrogen species (RNS) are the cause for the progression of several diseases, such as cardiovascular and neurodegenerative diseases, age-related diseases, alterations in the reproductive system, and cancer (Jacob et al., 2013; Pigini et al., 2022). In humans, the urinary biomarkers of oxidation of nucleic acids and proteins can be measured as the effect biomarkers of chemical exposures (Cavallo et al., 2021; Ghelli et al., 2021). They are formed as a result of the reaction of the hydroxyl radical •OH generated in cells as a consequence of the exposure to pollutants, dangerous substances, and lifestyles (smoking habits, nutrition, and alcohol) (Prasad et al., 2017). The hydroxyl radical • OH is characterized by a very high reactivity and easily reacts with the guanine (DNA base most susceptible to oxidation because of its low redox potential) bound to deoxyribose in DNA and ribose in RNA, releasing 8-oxo-7,8-dihydroguanine (8-oxoGua), 8-oxo-7,8-dihydro-2′-deoxiguanosine (8-oxodGuo), and 8-oxo-7,8-dihydroguanosine (8-oxoGuo) in urine (Cadet et al., 2012; Carrieri et al., 2019). In human urine, 8-oxoGua and 8-oxodGuo are derived from DNA repair mechanisms and also by the turnover of DNA damaged by oxidation (Chao et al., 2021; Tanaka and Chock, 2021) The concentrations of these indicators, measured in the urine, represent the share of oxidative damage suffered by the nucleic acids and the nucleotide pool; they also represent the share of oxidative damage the organism was able to repair spontaneously or following the activation of specific existing repair mechanisms.

Both O2•− and NO• react to form peroxynitrite ONOO−, a compound that reacts with tyrosine and an amino acid found in most proteins, respectively, giving 3-nitrotyrosine (3-NO_2_Tyr), which can be used as a marker of “nitro-oxidative” damage to proteins (Campolo et al., 2020).

In the present study, the occupational exposure to styrene of a group of shipbuilding industry workers was assessed by measuring, before and after the work shift, the urinary concentration of MA and PGA. The objective of this study was to assess the effects of the occupational exposure not only to styrene but also to the mixture of all the other chemicals used in the workplace, such as solvents and wood dusts. The oxidative stress was also evaluated by measuring urinary biomarkers. The results found in the exposed workers were compared to those of a group of healthy volunteers not occupationally exposed to chemicals.

## 2 Materials and methods

### 2.1 Studied population

The studied group included 17 male workers of a shipbuilding industry, who finished and assembled fiberglass-reinforced plastic parts of the boats. The population we used as control comprises 17 healthy volunteers without any exposure to chemicals. Only subjects living in very unpolluted areas were included in the control group. The volunteers were recruited among colleagues, their relatives, and students. The shipbuilding industry is located in a seaside town in central Italy. The produced boats are small- and medium-sized yachts that are assembled and refined. The present study was approved by the Ethical Committee of the Fondazione Policlinico Universitario Agostino Gemelli, Università Cattolica del Sacro Cuore, prot ID 5117 (nonprofit study), date 03/08/2022. All subjects provided their written informed consent to participate in the study and filled a questionnaire for the collection of the following information: gender, age, occupation, smoking status, and general health status. Workers were equipped with the most appropriate personal protective devices. The assessment of the exposure to chemical agents was carried out by the employer according to the Italian legislation, and results were in agreement with the occupational exposure limits.

This study can be defined as a non-interventionist/observational study, on the basis of the European Directive 2001/20/EC (2001); all experiments were conducted according to the Declaration of Helsinki, following the International Code of Ethics for Occupational Health Professionals published by the International Committee of Occupational Health (ICOH) (ICOH, 2014). Urine sampling was conducted during the medical surveillance of workers organized by the employer. The information gathered was used as aggregate data referring to the whole group of workers, with no risk of individual identification.

Table 1 shows the main subjects’ characteristics. Among workers exposed to styrene and other chemicals, different tasks were individuated, carpenter (seven workers), mechanical worker and welder (two workers), painter (three workers), and worker without any specific task (five workers). Furthermore, office employees (three workers) are exposed to chemicals as their workplace is very close to the industrial area.

**TABLE 1:** Characteristics of the subjects.

Subjects	Gender	Age (years) (min–max) mean (SD)	Smokers (%)	Average working hours/5 days	Urine sampling
Shipbuilding workers (n.17)	All males	(32–58) 45.75 (7.29)	35	40	Before and after work shift
Office employees (n.3)	1 male and 2 females	(44–66) 55.67 (11.06)	33	40	Before and after work shift
Volunteers (n.17)	All males	(45–67) 60.67 (5.31)	0	—	Spot sampling

### 2.2 Urine collection

All urine samples were collected on the same day in sterile polypropylene containers and divided into three aliquots, which were immediately transported to our laboratory and stored frozen at −25°C until analysis. In the workers’ urines, biomarkers were measured before and after the work shift, while for the control group, a spot urine sample was used. One aliquot of each urine sample was used to determine the urinary metabolites MA and PGA, one for the analysis of the oxidative stress biomarkers, and the third one for the determination of the creatinine concentration. The final concentration of all analytes was expressed as the ratio of the creatinine concentration in order to normalize their values with respect to the variability of urine dilution. This strategy was suggested by other authors (Heavner et al., 2006; Cone et al., 2009; Ozdemir et al., 2021). Urinary creatinine was determined by the Jaffè method using the alkaline picrate test with UV/VIS detection at 490 nm (Kroll et al., 1986). The samples having a creatinine concentration higher than 3 g/L or lower than 0.3 g/L have to be discarded, according to the recommendations of the ACGIH (ACGIH, 2016). The concentrations of creatinine lower than 0.3 g/L or higher than the upper limit of 3 g/L were not found in this study.

### 2.3 Chemicals and supplies

The analytical reference standards of DL mandelic acid (MA) and phenylglyoxylic acid (PGA) were purchased from Fluka (Sigma-Aldrich, Germany), and 8-oxoGua, 8-oxodGuo, and 8-oxoGuo were purchased from Spectra 2000 SRL (Rome, Italy). The deuterium-labeled internal standards ± mandelic-d_5_ acid (MA-d_5_) 99.4% and sodium phenyl-d_5_-glyoxylate 99.8% (PGA-d_5_) and the isotope-labeled internal standards (^13^C^15^N_2_), 8-oxodGuo, and (^13^C^15^N_2_) 8-oxoGuo were obtained from C/D/N Isotopes Inc. (Pointe-Claire, QC, Canada). The (^13^C^15^N_2_) 8-oxoGua (98%) was obtained from Cambridge Isotope Laboratories Inc. (Tewksbury, MA, United States). The 3-NO_2_Tyr was purchased from the Cayman Chemical Company (Ann Arbor, MI, United States) and 3-NO_2_Tyrd_3_ from Toronto Research Chemicals (Toronto, ON, Canada).

Glacial acetic acid 30% NH_3_, formic acid 50%, dimethyl sulfoxide, sodium hydroxide solution (50%–52% in water), and CHROMASOLV^®^ gradient grade 99.9% methanol and acetonitrile for HPLC/MS 99.9% carbon disulfide low benzene content were obtained from Sigma-Aldrich (Saint Louis, MO, United States). Purified water obtained from a Milli-Q Plus system (Millipore, Milford, MA, United States) was used for preparing the mobile phase and for diluting the samples.

Anotop 10LC syringe filter devices (0.2 m pore size and 10 mm diameter) were purchased from Whatman Inc. (Maidstone, United Kingdom). The Kinetex Polar C18 column 100 A (100 × 4.6 mm, 2.6 μm) and Kinetex Polar C18 column 100 A (150 × 4.6 mm and 2.6 m), supplied by Phenomenex (Torrance, CA, United States), were used throughout the study.

All urine samples were analyzed by liquid chromatography/tandem mass spectrometry (HPLC/MS-MS) using an API 4000 triple-quadrupole mass spectrometry detector equipped with a TurboIonSpray (TIS) probe (AB Sciex, Framingham, MA, United States) coupled to a Series 200 LC quaternary pump (PerkinElmer, Norwalk, CT, United States). Detection was carried out in the multiple reaction monitoring (MRM) mode, and parameters were optimized for the analytes by the automated infusion quantitative optimization procedure and then refined by flow injection analysis (FIA) using the pure standards. The 1.5 version of Analyst^®^ software (AB Sciex, Framingham, MA, United States) was used for instrument control and data analysis.

### 2.4 Determination of styrene metabolites (MA and PGA)

The MA and PGA quantitative analyses were carried out according to a validated analytical method (Paci et al., 2013). In brief, 1 mL urine samples were diluted with 1 mL of 2% acetic acid, added with 100 µL of internal standard (MA-d_5_ and PGA-d_5_) solution (10 mg/L) and filtered on a 0.2-μm syringe filter; 20 μL was injected into the HPLC-MS/MS system for quantitative analysis. Detection was carried out in the negative ion, MRM mode. The elution was carried out using a gradient of acetonitrile (phase A) and formic acid 0.2% (phase B), at a flow rate of 500 μL/min. Total run time was 10 min. The retention time of MA and its internal standard (MA-d_5_) was approximately 4.5 min, while for PGA and its internal standard (PGA-d_5_), the retention time was 4.0 min. The following m/z ion combinations (precursor→product) used both for identification and quantification were monitored, and the transitions were as follows: m/z −150.9→-107.3 for MA and -155.9→-112.0 for its internal standard MA-d_5_; −148.8→-105.1 for PGA and −153.8→-126.0 for its internal standard PGA-d_5_. The limit of detection (LOD) was 0.02 mg/L for MA and 0.015 mg/L for PGA, and the limit of quantification (LOQ) was 0.075 and 0.040 mg/L for MA and PGA, respectively. Accuracy was always higher than 82% and variability lower than 11% for both analytes. (Paci et al., 2013).

### 2.5 Biomarker oxidative stress (8-oxoGua, 8-oxodGuo, 8-oxoGuo, and 3-NO_2_Tyr)

The quantitative analysis of 8-oxoGua, 8-oxoGuo, 8-oxodGuo, and 3-NO_2_Tyr was determined according to the method proposed by Andreoli et al. (2010) with modifications in the sample thawing, dilution solvents, chromatographic column, and mobile phases. The samples were completely thawed in lukewarm water at approximately 37°C (Shih, Y-M., 2019), vortexed, and centrifuged at 10,000 x g for 5 min; the urine supernatant was added with internal standard and injected into the HPLC-MS/MS system. Detection was carried out in the positive ion, MRM mode. The elution was carried out using a gradient of methanol 9:1 v/v (phase A) and acetic acid 0.5% v/v (phase B) in water at a flow rate of 500 μL/min. Total run time was 18 min. The precursor→product ionic transitions monitored were 168.0→140.0 and 171.0 →143.0 for 8-oxoGua and its internal standard ((^13^C^15^N_2_) 8-oxoGua), 284.3→ 168.0 and 287.13 →171.1 for 8-oxodGuo and its internal standard ((^13^C^15^N_2_) 8-oxodGuo), 300.24→168.2 and 302.24→171.0 for 8-oxoGuo and its internal standard ((^13^C^15^N_2_) 8-oxoGuo), and 226.99 → 181.0 and 229.99 → 184.0 for 3-NO_2_Tyr and its internal standard (3-NO_2_Tyr d_3_). The concentrations of biomarkers and analytes were measured by using urine calibration curves. Eight different internal standards were used, spanning the range of interest. The concentrations were determined by measuring the ratio of the area of the analyte or biomarker to the area of the respective internal standard.

### 2.6 Statistical analysis

Statistical analyses were performed using an additional program of Microsoft Office Excel, Analysis Tool Pak (Microsoft Corporation, Redmond, WA, United States), after checking the log-normality of the distribution of data. Students’ *t*-test was used to analyze the difference between log-transformed values of the urinary concentrations, measured before and after the work shift in workers (paired *t*-test) and between exposed workers and non-exposed subjects (unpaired *t*-test). The linear bivariate correlation between variables was also determined on the log-transformed values. In all tests, *p*-value lower than 0.05 was considered as statistically significant.

Multivariate analyses were carried out with statistical software R (R version 4.2.1 (2022-06-23)). The sparse PLS-DA (sPLS-DA) technique was applied with the aim of determining the combination of variables, maximally discriminating between the exposed from the control group and discriminating different working tasks. sPLS-DA permits to project the data in a subspace containing only the variables that explain the major fraction of variances. If the maximum number of variables is set, n, starting from an initial space dimension N, the principal components (PCs) will be created as a linear combination of the n variables maximally contributing in explaining the variance. For sPLS-DA, a package of R in the library mixOmics was used. A factor variable “task” was introduced and was characterized by four levels: “c” for carpenters, “m” individuating welders and mechanical workers, “p” for painters, and “w” for a generic worker. The groups “c” and “m” were subsequently gathered in a unique group named group 1, and the painter and generic worker, groups “p” and “w,” were grouped in a unique group named group 2.

sPLS-DA was applied to the exposed workers belonging to the two groups previously described in order to individuate the variables and to better discriminate different tasks. All the oxidative stress variables, 8-oxoGua, 8-oxoGuo 8-oxodGuo, and 3-NO_2_Tyr, and the dose biomarkers, i.e., the styrene metabolites, MA and PGA, were used in this analysis. Another analysis was performed only on the oxidative stress variables in order to study what combination better discriminates exposed from non-exposed subjects.

## 3 Results and discussion

In this study, the analyte concentration levels show the descriptive statistics expressed in terms of mean with the standard deviation, 5th, 50th or median, and 95th percentile, as reported in Table 2 for the urinary metabolites MA and PGA expressed in mg/g creatinine.

**TABLE 2:** Concentrations of the urinary metabolites MA and PGA in the exposed subjects: descriptive statistics.

Analytes (mg/g creatinine)	MA		PGA		MA + PGA	
**Shipbuilding workers (*n* = 17)**	***BS***	***AS***	***BS***	***AS***	***BS***	***AS***
**Mean (SD)**	**4.29 (6.84)**	**26.19 (55.08)**	**3.86 (5.20)**	**10.38 (17.36)**	**8.15 (11.75)**	**36.51 (72.16)**
**5**th **percentile**	0.19	1.66	0.14	0.47	0.62	2.55
**50**th **percentile**	1.25	7.46	1.49	3.16	3.73	9.49
**95**th **percentile**	13.92	92.36	12.46	34.32	26.38	124.61
**Office employees (*n* = 3)**	***BS***	***AS***	***BS***	***AS***	***BS***	***AS***
**Mean (SD)**	0.23 (0.10)	0.020 (0.11)	0.04 (0.02)	0.14 (0.20)	0.27 (0.12)	0.33 (0.31)
**5**th **percentile**	0.14	0.12	0.02	0.02	0.17	0.14
**50**th **percentile**	0.22	0.15	0.04	0.02	0.26	0.17
**95**th **percentile**	0.32	0.31	0.05	0.33	0.38	0.64

BS, before shift; AS, after shift. In bold are the means significantly different in the comparison before and after the work shift.

The mean values of MA and PGA measured after the work shift and their sum are higher than those measured before (*p* = 0.00007; *p* = 0.025; and *p* = 0.0003, respectively) for the shipbuilding workers. However, the sum of the two metabolites at the end of the shift is much lower than the ACGIH BEI of 400 mg/g creatinine (ACGIH, 2020). The urinary metabolites of office employees are very low, as expected, as they should not be exposed to styrene, and there is no statistically significant difference between before and after the work shift. The high metabolite levels at the beginning of the work shift is an indication of a possible slowdown of styrene metabolism caused by the co-exposure to other organic solvents and, in particular, acetone, which is used as a cleaning agent for the tools, as observed in a previous study (Bonanni et al., 2015).

Table 3 shows the descriptive statistics for the four oxidative stress biomarkers measured in the same urine samples, expressed in µg/g creatinine. The values are compared to those of 17 healthy volunteers, all males, involved in this study as a control group.

**TABLE 3:** Concentrations of the oxidation biomarkers in all subjects.

Biomarkers (µg/g creatinine)	8-oxoGua		8-oxodGuo		8-oxoGuo		3-NO_2_Tyr	
**Shipbuilding workers (*n* = 17)**	***BS***	***AS***	***BS***	***AS***	***BS***	***AS***	***BS***	***AS***
**Mean (SD)**	**51.70 (38.85)**	**76.35 (42.73)**	**22.31 (8.54)**	**19.60 (7.94)**	7.12 (9.31)	6.76 (5.54)	20.02 (12.18)	18.78 (13.89)
**5**th **percentile**	6.77	16.16	12.41	10.21	1.93	1.45	6.12	6.72
**50**th **percentile**	40.71	67.52	21.33	18.46	3.91	4.28	19.08	15.45
**95**th **percentile**	125.57	144.38	39.49	31.63	19.27	18.25	42.81	48.35
**Office employees (*n* = 3)**	***BS***	***AS***	***BS***	***AS***	***BS***	***AS***	***BS***	***AS***
**Mean (SD)**	26.48 (16.20)	17.74 (14,91)	37.94 (6.54)	35.16 (4.83)	5.75 (2.42)	3.88 (0.83)	8.82 (4.07)	10.52 (3.25)
**5**th **percentile**	14.49	4.12	33.21	30.55	3.84	3.06	5.63	8.33
**50**th **percentile**	20.94	18.33	35.53	36.08	5.14	4.33	7.73	9.07
**95**th **percentile**	42.35	30.94	44.37	39.12	8.09	4.38	12.76	13.73
**Volunteers (*n* = 17)**								
**Mean (SD)**	16.97 (15.07)		7.16 (5.41)		9.54 (4.22)		13.66 (7.20)	
**5**th **percentile**	1.78		1.76		3.97		4.37	
**50**th **percentile**	13.04		5.77		9.41		15.98	
**95**th **percentile**	52.56		17.75		15.51		22.76	

BS, before shift; AS, after shift. In bold are the means significantly different in the comparison before and after the work shift.

In terms of all the biomarkers considered in this study, both styrene metabolites and oxidative stress biomarkers, no statistically significant differences were found between smokers and non-smokers in the exposed group, both at the beginning and end of the work shift. We interpreted this finding to be related to the fact that the exposure to toxic chemicals, coming from the working environment, largely exceeds the exposure that could be related to the smoking habit.

For what concerns the biomarkers of oxidative stress, the statistical comparison performed by the paired *t*-test shows that the mean concentration values of 8-oxoGua in the shipbuilding workers are higher after the work shift than before. A statistically significant difference was also found for 8-oxoGua and 8-oxodGuo values that are higher in the shipbuilding workers than in the control group, both when measured before and after the work shift. The values of 8-oxoGua and 3-NO_2_Tyr of the shipbuilding workers are higher than those of the office employees.

With the aim of facilitating reading of the results, we reported in Table 4 the ratio between the average concentrations of the oxidative stress biomarkers in exposed workers and in the group of healthy volunteers.

**TABLE 4:** Normalization within different populations.

	8-oxoGua		8-oxodGuo		8-oxoGuo		3-NO_2_Tyr	
**Shipbuilding workers (mean)/office employees (mean)**	***BS***	***AS***	***BS***	***AS***	***BS***	***AS***	***BS***	***AS***
	1.95	4.30	0.59	0.56	1.24	1.74	2.25	1.79
**Shipbuilding workers (mean)/volunteers (mean)**	***BS***	***AS***	***BS***	***AS***	***BS***	***AS***	***BS***	***AS***
	3.05	4.50	3.12	2.74	0.75	0.71	1.47	1.37
**Office employees (mean)/volunteers (mean)**	***BS***	***AS***	***BS***	***AS***	***BS***	***AS***	***BS***	***AS***
	1.56	1.05	5.30	4.91	0.60	0.41	0.65	0.77

The log-correlations (Pearson’s) among the exposure biomarkers (the styrene metabolites) and the effect biomarkers (the oxidative stress biomarkers) were also studied, and the results are reported in Table 5. For the shipbuilding workers, the correlation was studied separately before and after the work shift, while for the office employees, the values of MA, PGA, and their sum measured before and after the work shift were combined as there was no statistically significant difference between the two series.

**TABLE 5:** Pearson’s correlation of exposure and effect biomarkers (calculated on log-transformed values).

**Shipbuilding workers (before shift)**				
	**8-oxoGua**	**8-oxoGuo**	**8-oxodGuo**	**3-NO**_**2**_ **Tyr**
**MA**	−0.02	−0.12	−0.41	−0.19
**PGA**	0.01	0.06	−0.30	−0.29
**∑MA + PGA**	0.03	−0.05	−0.39	−0.21
**Shipbuilding workers (after shift)**				
	**8-oxoGua**	**8-oxoGuo**	**8-oxodGuo**	**3-NO**_**2**_ **Tyr**
**MA**	0.22	0.27	−0.60	−0.02
**PGA**	0.31	0.27	−0.30	0.03
**∑MA + PGA**	0.21	0.29	−0.56	−0.03
**Office employees (pooled data)**				
	**8-oxoGua**	**8-oxoGuo**	**8-oxodGuo**	**3-NO**_**2**_ **Tyr**
**MA**	−0.44	0.79	−0.02	0.12
**PGA**	−0.83	0.29	−0.02	−0.05
**∑MA + PGA**	−0.67	0.56	−0.02	0.04

In bold are the means significantly different in the comparison before and after the work shift.

The results show that the urinary 8-oxoGuo is positively correlated in shipbuilding workers with MA, PGA, and ∑MA + PGA. This result confirms the results observed in a previous study (Manini et al., 2009), showing high levels of urinary 8-oxoGuo and correlating with internal exposure metabolites (∑MA + PGA), in styrene-exposed workers. Another study on the levels of urinary biomarkers of oxidatively generated damage to DNA and RNA in different groups of workers compared to the general population concluded that the urinary 8-oxoGuo, which is related to RNA oxidation, seems to be the most suitable biomarker to detect short-term, reversible effects of exposure to dangerous chemicals (Tranfo et al., 2019) and metals (Buonaurio et al., 2021. Furthermore, in agreement with other recent works (Broedbaek et al., 2011; Kjær et al., 2017; Gan et al., 2018), urinary 8-oxoGuo may be associated with an increase in mortality, considering it as a potential biomarker to identify individuals at high risk of developing age-related diseases.

The correlation coefficients between styrene metabolites and urinary 8-oxoGuo are not very high, and they do not reach statistical significance. On the other hand, we should consider that the workers of the shipbuilding industry are exposed not only to styrene but also to other hazardous chemicals, such as organic solvents contained in paints and wood dust that could contribute to oxidative stress. In office employees, MA and the sum MA + PGA are also well correlated to the urinary 8-oxoGuo, if we include the concentrations measured before the work shift, but this is not true for PGA. Therefore, the oxidative stress cannot be derived from styrene exposure only but from different sources. In fact, mandelic acid can be found in some foods (mainly almonds), but it is also used in cosmetics and as an antibiotic. The very low number of office employees does not permit drawing conclusions regarding the association between MA and PGA and the urinary concentration of 8-oxoGuo in this group.

### 3.1 sPLS-DA discrimination by task

**FIGURE 1:**
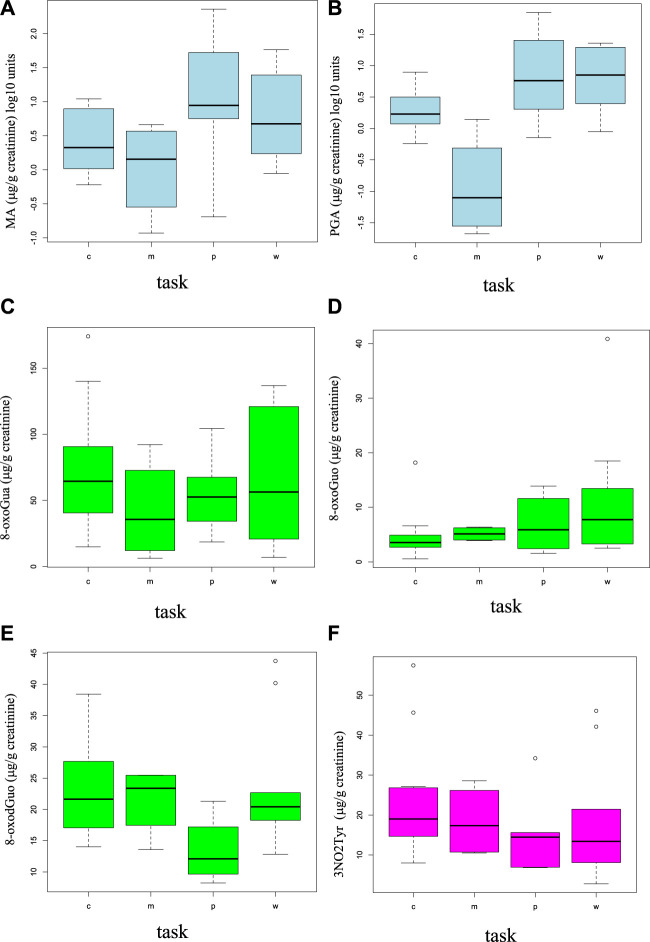
Boxplots showing the distributions (median in the center and the first and fourth quartiles as rectangle delimiters) of the styrene dose exposure biomarkers (MA and PGA **(A, B)**) and the oxidative stress biomarkers [8-oxoGua **(C)**, 8-oxoGuo **(D)**, 8-oxodGuo **(E)**, and 3-NO_2_Tyr **(F)**] in different working tasks (“c” = carpenter, “m” = welder and mechanical worker, “p” = painter, and “w” = generic worker).

The distributions of the dose and oxidative stress biomarkers have been studied separately for the different working tasks: carpenters (*n* = 7), mechanical workers and welders (*n* = 2), painters (*n* = 3), and workers without a specific task (*n* = 5). Figures 1A–F show the boxplots for these four groups for each biomarker.

The painters are the workers having the maximum average levels of MA and PGA urinary concentrations. The maximum average concentrations of 8-oxoGua and 8-oxodGuo were found in carpenters. Due to the poor number of workers in different task groups, the workers were grouped in only two groups, based on their styrene exposure. Group 1, including carpenters, welders, and mechanical workers (*n* = 9), has the lowest styrene exposure, while group 2, including painters and generic workers engaged in the assembly of parts of the boats (*n* = 8), has a higher styrene exposure.

Tables 6, 7 show the urinary concentrations of the dose and oxidative stress biomarkers for the two groups.

**TABLE 6:** Concentrations of the urinary metabolites MA and PGA in groups 1 and 2.

Workers group	MA (mg/g creatinine)							
	*BS*				*AS*			
		Percentile				Percentile		
	Mean (SD)	5th	50th	95th	Mean (SD)	5th	50th	95th
**1**	**1.96 (3.09)**	0.31	0.94	6.75	**5.52 (3.32)**	1.89	4.60	10.29
**2**	**6.90 (9.01)**	0.44	4.55	21.64	**49.30 (75.92)**	2.68	20.09	169.51
	**PGA (mg/g creatinine)**							
**1**	**1.40 (1.12)**	0.08	1.18	3.21	**2.61 (2.37)**	0.25	1.70	6.48
**2**	**6.63 (6.62)**	0.78	4.83	16.99	**19.13 (22.74)**	1.34	13.69	54.45
	**MA + PGA (mg/g creatinine)**							
**1**	**3.36 (3.39)**	0.45	2.33	8.72	**8.13 (5.42)**	2.40	6.53	16.78
**2**	**13.53 (15.48)**	1.22	9.68	38.62	**68.43 (98.33)**	4.85	33.78	223.06

BS, before shift; AS, after shift. In bold are the values of the means significantly different in the comparison between before and after work shift.

**TABLE 7:** Concentrations of the oxidative stress biomarkers in groups 1 and 2.

Workers group	8-oxoGua (µg/g creatinine)							
	*BS*				*AS*			
		Percentile				Percentile		
	Mean (SD)	5th	50th	95th	Mean (SD)	5th	50th	95th
**1**	55.07 (40.82)	9.74	52.64	118.96	77.05 (45.68)	23.17	84.45	141.39
**2**	47.91 (38.92)	11.00	37.47	109.97	75.56 (42.29)	23.09	65.44	131.34
	**8-oxodGuo (µg/g creatinine)**							
**1**	23.51 (7.17)	15.24	23.07	34.57	20.90 (5.85)	13.84	20.22	28.76
**2**	20.95 (10.19)	10.87	19.96	36.36	18.14 (10.02)	9.11	15.88	33.47
	**8-oxoGuo (µg/g creatinine)**							
**1**	**3.76 (1.87)**	1.25	3.70	6.43	5.46 (5.01)	1.68	4.10	13.46
**2**	**10.91 (12.78)**	2.49	6.71	31.42	8.21 (6.08)	1.89	7.11	16.72
	**3-NO**_**2**_ **Tyr (µg/g creatinine)**							
**1**	22.02 (10.90)	10.17	20.47	38.22	21.07 (15.16)	8.82	17.26	45.90
**2**	17.77 (13.88)	4.24	12.92	39.34	16.21 (12.81)	6.45	14.94	35.87

BS, before shift; AS, after shift. In bold are the values of the means significantly different in the comparison between before and after work shift.

The mean values for MA, PGA, and their sum are statistically higher after the work shift in both groups and in group 2 that comprises painters and generic workers with respect to group 1 (*t*-test, *p* < 0.05, data in bold).

Regarding the oxidative stress biomarkers, only 8-oxoGuo is higher in group 2 than in group 1, but only before the work shift (data in bold). This can be interpreted as a long half-life of the considered biomarkers that is still excreted in the morning after the exposure to chemicals. The number of subjects is small, so conclusions cannot be drawn regarding this finding.

**FIGURE 2:**
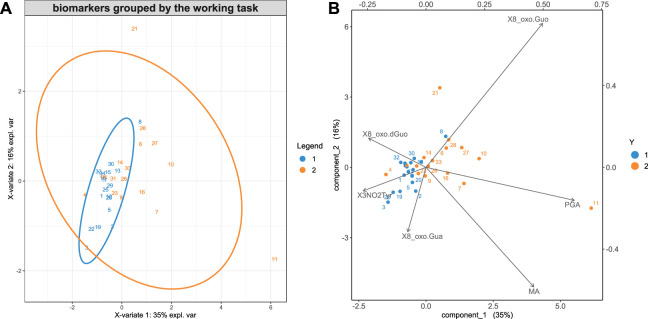
**(A)** The cases are plotted in the principal component 2d plane individuated by sPLS-DA to find the maximum discrimination between group 1 and 2. **(B)** The variables are also represented in the principal component plane.

Figure 2, A and B, shows the results of the sPLS-DA analysis discriminating group 1 and group 2 (all data for both BS and AS), respectively.

Figure 2 shows that the workers of group 2 have a larger exposure to styrene, as they excrete a higher concentration of styrene metabolites, MA and PGA, in their urine. In group 2, the 8-oxoGuo is higher than in group 1, which is characterized by higher concentrations of 8-oxoGua and 8-oxodGuo. A previous study suggested that 8-oxoGuo could be the most sensitive biomarker to the short-term, reversible effects of exposure to chemical agents even under conditions that could be considered safe, as the styrene exposure below the occupational exposure limits (Tranfo et al., 2019). 8-OxodGuo, derived from damage caused to the DNA, both if repaired and/or not repaired**,** is typically higher when the exposure to carcinogenic agents occurs, which, in the case of group 1, formed by carpenters and welders, could be identified with wood dust and welding fumes, respectively.

### 3.2 sPLS-DA discrimination between workers and the control group

The results of the exposed workers (*n* = 34, before and after the work shift) were compared to those of the 17 volunteers shown in Table 1. This choice was supported by the fact that the three employees shared a common environment with the workers and, consequently, were also exposed to different airborne pollutants, and therefore, they cannot be considered non-exposed subjects. In addition, both in the group of exposed workers and in that of office employees, a certain percentage (approximately 30%) of smokers were present contributing to the oxidative stress. The non-exposed, healthy volunteers are also non-smokers, making the discrimination between the exposed and non-exposed more effective.

**FIGURE 3:**
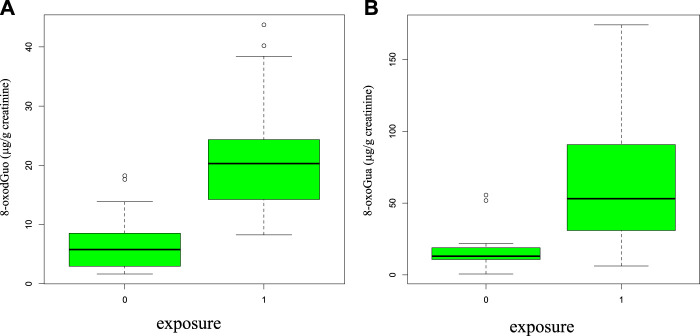
Boxplot representing the statistical distributions (median in the center and the first and fourth quartiles as rectangle delimiters) of 8-oxodGuo **(A)** and 8-oxoGua **(B)** in the group of exposed (group = 1, shipbuilding workers, all tasks) and control subjects (group = 0, 17 healthy volunteers).

The distributions of 8-oxoGua and 8-oxodGuo in the groups of exposed and control subjects are shown in the boxplots of Figure 3, A and B, as, of the four biomarkers related to the oxidative stress, only these two were found differently concentrated at a statistically significant level between the two groups (*p* = 5.57*10^-7 and *p* = 5.77*10^-9 for 8-oxoGua and 8-oxodGuo, respectively).

**FIGURE 4:**
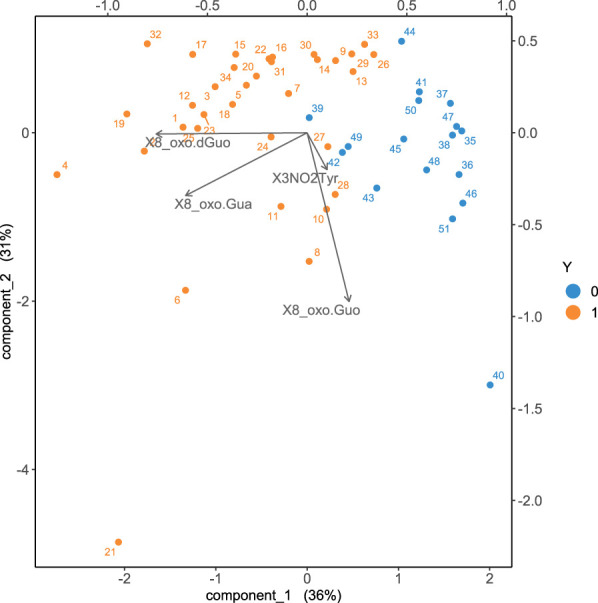
Biplot obtained with sPLS-DA discriminating the exposed from the control group. The cases of the non-exposed subjects are represented in blue, whilst the exposed workers are represented in orange. The arrows represent the original variables projected in the 2d plane principal component space.

Including all the four oxidative stress biomarkers into the sPLS-DA analysis, a good discrimination of the two aforementioned groups, exposed and controls, is obtained as shown in Figure 4.

**FIGURE 5:**
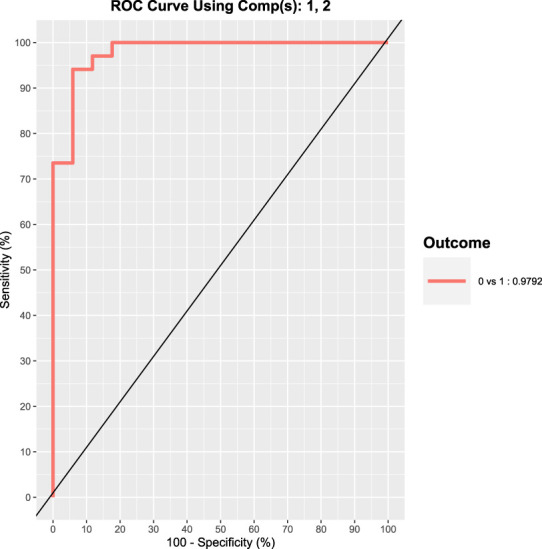
ROC curve representing the statistical power of the discrimination between exposed (17 shipbuilding workers) and control subjects (17 healthy volunteers) based on the oxidative stress biomarker excretion.

As it can be appreciated by visually inspecting the plot in Figure 4, the exposed subjects are characterized by increased values of 8-oxoGua and 8-oxodGuo biomarkers whilst the concentrations of 8-oxoGuo are larger in the controls. The statistical power of the discrimination between exposed and controls is quantified by means of the ROC curve, as shown in Figure 5.

This curve shows a very high sensitivity and specificity in discriminating controls from exposed subjects, measured by the area under curve that is approximately 98%. Further studies will be devoted to the development of a test based on 8-oxoGua and 8-oxodGuo in order to discriminate, at high levels of sensitivity and specificity, subjects exposed to carcinogenic agents from subjects who are not exposed to carcinogenic agents.

### 3.3 Limitations of the study

This study is strongly limited by the small number of subjects. In particular, it is difficult to compare between the different working tasks with such a small sample size. The limitation of this study is strictly related to the production structure as Italy is dominated by small and medium enterprises characterized by handcrafted features. This work reflects this structure. The small number of subjects was counteracted by a very precise evaluation of dose metabolites in combination with oxidative stress biomarkers. In any case, it must be stressed that what was found is based on a small sample and must be confirmed on a larger one.

## 4 Conclusion

This study aimed to measure the levels of oxidative stress biomarkers in workers exposed to styrene in a shipbuilding industry in which workers finish and assemble fiberglass plastic parts of the boats. In order to assess styrene exposure, the concentrations of urinary styrene metabolites, MA and PGA, were measured both at the beginning and end of the work shift.

When the oxidative stress biomarkers were compared in exposed workers and in volunteers without any professional exposure to chemicals, a significant discrimination was obtained. The area under the ROC curve, discriminating exposed from control subjects, was approximately 98%. The discrimination was found mainly due to the concentrations of 8-oxoGua and 8-oxodGuo that are biomarkers known to be related to nucleic acid damage.

The stratification of results according to different working tasks showed larger exposure to styrene in painters and generic workers, who show significantly higher concentrations of 8-oxoGuo and relatively lower concentrations of 8-oxoGua and 8-oxodGuo, with respect to the group of carpenters and welders.

The oxidative stress biomarker profile in the shipbuilding industry is quite complex due to the complexity of the working tasks, involving different exposure factors, that could contribute to the increase in the oxidative stress biomarker excretion in the urine. In fact, the workers are exposed not only to styrene but also to paints, wood dust, coming from the refining of the parts of the boat, and welding fumes. Even if the oxidative stress biomarkers are sensitive to the occupational exposure to chemicals and could be used as an early warning for their health effects, a thorough analysis of the exposure sources is necessary to determining the level of oxidative stress induced by the exposure to different xenobiotics.

## Data Availability

The raw data supporting the conclusion of this article will be made available by the authors, without undue reservation.
